# Ecolabels and the economic recession

**DOI:** 10.1371/journal.pone.0294167

**Published:** 2023-12-04

**Authors:** Jibonayan Raychaudhuri, Ada Wossink

**Affiliations:** 1 School of Economics, University of East Anglia, Norwich, England, United Kingdom; 2 Department of Economics, University of Manchester, Manchester, England, United Kingdom; Huanggang Normal University, CHINA

## Abstract

We examine the effect of the 2008 economic recession on consumers’ observed expenditures for eco-labelled grocery products. Traditional price theory predicts that consumers change their spending during an economic downturn and we would expect the sales share of eco-labelled products to fall since these are relatively more expensive than non-labelled products. We use supermarket loyalty card data from the UK and show that the recession had widely different effects on the expenditure share of different eco-labelled grocery products. We confirm, empirically, that expenditure shares on organic products declined over the time period under study but the expenditures share for fair-trade products increased over the same period. We evaluate alternative models of decision making to explain our results, *viz*., a salience model and a model of reputation signalling. We find that both of these models give a plausible explanation of our empirical results.

## 1 Introduction

When consumers buy a product, they make their decision whether to buy the product based not just on the price and consumptive characteristics of the product. They also consider how the product was produced, i.e., the extent in which environmentally sustainable and ethical production practices were employed. Consumers prefer these socially valued public good attributes in their products much like they prefer any other desirable product quality attribute in market goods. Such attributes need not have any effect on consumptive characteristics: for instance, ‘dolphin-safe’ tuna tastes no different than conventional tuna, but may be more valued by consumers, nonetheless [1].

Consumer preference for socially valued public good features of a product has led to the birth and growth of a new market for (so called) “eco-labelled” products. Eco-labelling initiatives are positioned at an interface between shifting production and consumption demands (and the relation between these demands). Eco-labels address a problem of asymmetric information. Typically, consumers are not able to experience a public good attribute because this is a characteristic of the production process and not of the good itself. The aim of eco-labels on products is to inform buyers about a product’s superior public good attributes: they provide information to consumers that the product has been produced in an ethical and/or environmentally friendly way. Thus, simply stated, eco-labelling can circumvent market inefficiencies by making the information initially held by the firm also available to the consumer. This removal of information asymmetry is clearly beneficial to consumers as their choices will be more closely in line with preferences. Firms that produce goods with desirable public good attributes also gain. If buyers are willing to pay more for the public good attributes of a product, then these products can command a higher price or have increased sales in the market. Eco-labelling thus offers the possibility of improved market access and/or the capture of a greater proportion of the value of production as the higher prices obtained offset more accurately the costs of production [2, 3]. The consumer is provided with the information on these public good characteristics mainly through voluntary eco-labelling schemes.

Eco-labels are now seen as an effective policy tool to change consumption to more sustainable levels. The effectiveness of eco-labels as a policy tool to achieve environmental goals is addressed in a growing literature [4, 5]. The theoretical research has focused on examining the design and efficacy of different labelling schemes, the effects of labelling in production and trade, and has modelled eco-friendly consumer behaviour (for example, [6–11]). The empirical research has focused on finding consumers’ willingness to pay for different labels, in particular, for “green” or ethical product labels (see [12] for a review of the literature). Consumer choice for ethical products in a market context has received little attention. Most of the existing empirical evidence has come from work with data collected from surveys on hypothetical choices or from incentivised experimental settings (for example, see [13]), rather than from data based on actual observed purchases (a notable exception is [14]). Surveys and laboratory experiments are useful for eliciting well-informed, thoughtful preferences. But, in these settings, an upward bias for WTP is likely because of the artificial environment and limited number of products examined [4]. Hence situations under which these eco-labels can command a price premium or have increased sales in a market set-up are far from fully understood.

Our paper looks at the effect of the recession that started in September 2008, in the United Kingdom (UK), on observed consumer expenditure for eco-labelled food products. These are products that differ in socio-economic quality. Our research is motivated by an interesting observation in trade reports. These reports claim that organic grocery sales in the UK have fallen, whereas fair trade sales have held up, during this economic downturn [15, 16]. We know that during an economic downturn, consumers become more price sensitive. Both organic and fair trade varieties of grocery products are usually more expensive than their non-labelled varieties. So we would expect the sales shares for grocery products in both of these eco-labelled categories to fall relative to the sale of conventional groceries. In Canada and the US, Fair Trade consumers did decrease their purchases [16]. We think that this prediction is more likely to be observed in the data for grocery products, as consumers do not have the option to hold-off purchases of groceries.

The first contribution of our paper is to look at the trends in consumer expenditure on eco-labelled grocery products during a recession when consumers face an income shock. We use supermarket loyalty card data for a range of food products sold under different eco-labels (such as, organic, fair trade and carbon-labelled) from a noted super market chain in the UK for our study. We employ data-driven methods to investigate how the recession impacted consumers’ aggregate purchase behaviour. Then we consider an econometric specification that looks at the difference, pre and post recession, in the purchases of various eco-labelled products (at a disaggregated level). We find that the share of consumer expenditure on fair trade products seems to hold up during the recession while the share of organic products seems to fall.

The second contribution of our paper is to compare behavioural explanations for our empirical findings [17]. We evaluate two alternative models of decision making that give a plausible explanation for our results. We consider a model of salience applied to consumer choice [18] and a model of reputation signalling where image concerns and the behaviour of other consumers affect consumer choice [19, 20]. We find that both of these models explain the features observed in our data.

The remainder of this paper is organized as follows. Section Background, Data and Summary Statistics provides background on the main eco-labels investigated in the paper. This section is followed by a detailed description of the scanner data that we use in our analysis. Section Results reports the empirical results. Section Alternative Underpinnings of Eco-labelled Consumption discusses the different psychological models of decision making and evaluates their predictions against our empirical results. Section Discussion discusses some policy implications of our work. Section Conclusion concludes.

## 2 Background, data and summary statistics

### 2.1 Background on organic and fairtrade food products

Eco-labels highlight specific sustainable aspects of the production process for a good [21]. These aspects can be further divided by the three “dimensions” of sustainability, namely (*i*) environmental aspects, such as protection of water, soil, animal welfare, biodiversity as well as conservation and enhancement of landscapes; (*ii*) economic aspects, such as, fair prices and contracts for farmers and workers in the developing world; (*iii*) social aspects, such as, fair, safe and equitable working conditions and child free labour.

The three main categories of eco-labels in the food market, those for organic, fair trade and carbon-labelled products, differ in the emphasis that they place on these three aspects of sustainability. The Eco-label Index database lists over 450 widely recognised eco-labelling program operating in 197 countries and 25 industry sectors. This includes 148 eco-labels on food. See www.ecolabelindex.com. This database is currently the most exhaustive database on eco-labels that is available for research purposes. Organic labels focus on the method of production; organic food is food which is produced using environmentally friendly and animal friendly farming methods. These methods are legally defined. In the EU, any food product sold as ‘organic’ falls under the EU regulations 834/2007 and 889/2008. See https://www.soilassociation.org/. Fair-trade labels focus primarily on the economic aspect by offering higher prices to producers (usually in developing countries), thereby improving their long-term living conditions. See the definition of fair trade adopted by the international fair trade movement in 2001 at https://www.newefta.org/. Fair trade products are certified by labelling organisations such as Max Havering or Fair trade International (FLO). Details about the certification standards are given at https://www.fairtrade.org.uk. Some Fair trade products also volunteer information on how the product (such as coffee or chocolate) was grown organically and so these products also bear an organic label, but in general this information is not required. Another point of difference between organic and fair trade labels is with respect to the use of logos (or exposition). For fair trade food there is a common and distinctive logo used in almost all markets. The exceptions are Mexico and USA. In contrast there is no universal organic logo (see for e.g., [22]). Finally carbon labelling shows the amount of emissions of six greenhouse gases over a product’s life cycle. This label is designed to inform consumers about the embedded carbon content of a product and to allow them to compare products, so that they can choose the product with the smallest carbon footprint [23].

As mentioned earlier, eco-labelling systems use the market to provide public good attributes. However, these public good characteristics of eco-labelled products may be extended/combined with other private characteristics when the product is finally presented to the consumer. Consumers might infer subjective quality beliefs from a label in line with a halo effect (see [24]). For example, [25, 26] find that health concerns are an important primary motive for organic food consumption even without convincing proof that organic food is better for health.

Studies on the consumption of fair trade products are few, but here again private values—in particular quality attributes such as brand and flavour—play a role (see [27]). We will return to the role that these private values play in a consumer’s buying decision in section Alternative Underpinnings of Eco-labelled Consumption of this paper.

As mentioned above, our paper is inspired by an intriguing observation in trade journals. Retail sales of fair trade in the UK was reported to have increased by 14 percent in 2009 relative to the year before [28]. Over the same period, retail sales of organic products in the UK decreased by 12.9 percent [29]. It is tempting to attribute the decline in organic food products to the recession. However, the increase in fair trade sales trade contradicts any such simple explanation. Traditional price theory predicts that consumers change their spending during an economic downturn as they become more price aware (e.g., [30]). This theory assumes that consumer taste does not change during a recession but, as they are faced with smaller budgets, they have less to spend on luxuries and therefore allocate larger share of their budgets to more essential categories. Thus, we would expect the UK sales of both organic and fair trade foods to decreased since these are commonly more expensive than non-labelled food goods. Our empirical analysis uses supermarket scanner data to first of all statistically verify the findings from the trade reports for the 2008 economic recession. We analyse aggregate expenditure trends by category of eco-labelled food products and investigate the trend over the same period at the level of the individual consumer and by consumer groups. We find that our analyses corroborate the difference in UK expenditure trends by eco-labelled category over the period of the recession. Given our empirical results, the traditional price theory perspective offers no explanation. This finding is in line with for example [31] who point out that there is limited empirical evidence for the assumption that consumer taste remains unchanged regardless of economic conditions. Given this outcome, we then turn to alternative interpretations for our main empirical findings. We consider a model of salience applied to consumer choice [18] and, drawing on the recent literature of consumers’ behavioural motives, we postulate that consumers might derive indirect utility from the public good attribute of a food product [32, 33]. We find that both models explain the patterns observed in our data.

### 2.2 Data

Our empirical exercise uses revealed preference (scanner) data on food consumption recorded at a leading UK retailer. Our data represents a random sample of 60,000 (club) card account holders of this supermarket chain covering nationwide sales. Our empirical analysis focuses on weekly observations starting from the financial week 17 of 2007 and extending up to (and including) the financial week 15 in 2009. Thus our data covers a period of 104 weeks (36 weeks in 2007, 52 weeks in 2008 and 16 weeks in 2009). We note that the weeks mentioned above are not actual calendar weeks but these are financial weeks of the supermarket chain in question. In the United Kingdom, the financial year runs from 1 April of a year to 31 March of the next year. We have data starting from calendar week 13 in 2007 (starting March 26, 2007 to April 1, 2007). So calendar week 13 corresponds to the supermarket chain’s financial week 1. We use this “mapping” of calendar to (the supermarket’s) financial weeks to transfer important dates. For example, Lehman Brothers filed for Chapter 11 bankruptcy protection on September 15, 2008 which was calendar week 38 in 2008 and week 61 in our data.

Our random sample of 60,000 club card account holders comprises of consumers who have been monitored periodically and who have had information collected on them through the Shopper Thoughts Panellist surveys. So, we call our sample the panellist sample. In our panellist sample, we have repeated observations for club card account holders recording the various grocery item purchases that they have made (over the time period of our analysis) in this supermarket. Our dataset is limited to only one supermarket and we cannot observe whether there may have been systematic changes in market shares of certain products or in the buying behaviour of customers as they shift to making purchases from other supermarkets. However, we do note that the supermarket chain we have data for is a major supermarket chain with a market share of over 31 percent in the UK in 2008 (TNS Worldpanel). Our data is a random sample from all card account holders (around 16.5 million club card account holders) of this supermarket chain covering nationwide sales.

For the panellist sample, we have item level transaction information of expenditure on the purchase of various “ethical” products (products that are labelled into one of the groups like organic, fair trade, etc.) and common products for the 104 weeks. In addition to the transaction information, we also have some additional demographic information on these consumers. These include information on the “life stage” of the club club holder such as whether the club card holder is a pensioner, or has a young family etc. The life stage classification is a six-stage life style segmentation with the following groups: Older Adults (OA), OLder Families (OF), Pensioners (PE), Young Adults (YA), Young Families (YF), Other (OT).We note that for the panellist sample we have information at the consumer level.

From the panellist sample of 60,000 records, we aggregate weekly expenditures for each of the 104 weeks in our sample, for each of the following 5 (broad) classes of products: “organic”, “fair trade”, “carbon” and “other”. The first three terms are self-explanatory. The last category “other” is expenditure on items that do not belong to any of the aforementioned three categories.

### 2.3 Summary statistics

We start our analysis by looking at the trends in the aggregate data. This is data in S1 File. Aggregate expenditure data. We report summary statistics for this aggregated data set in Table 1.

**Table 1:** Summary statistics: Weekly consumer expenditure (two years)^a^.

Variable	Mean	Std. Dev.	Min.	Max.	N
Total Amount Spent	595154.731	43596.953	386578	793943	104
Amount Spent on Organic	58116.917	4792.906	37615.398	71768	104
Amount Spent on Fairtrade	12915.078	2989.469	4303.13	18760.1	104
Amount Spent on Carbon	17557.151	4219.054	13449.6	42363.898	104
Amount Spent on other products	504746.192	36844.508	327066	665978	104

^a^ The above table shows summary statistics for the *weekly* expenditure on each major eco-labelled product category—organic, fair trade, carbon, carbon-fair trade and the category, “other”. All figures shown are in pounds (£) and in levels. These statistics are compiled from scanner-level (revealed preference) data on food consumption recorded at a leading UK retailer with a market share over 31 percent in 2008. Our empirical analysis focuses on weekly observations for financial week 17 of 2007 up to and including financial week 15 in 2009. Thus the date covers a period of 104 weeks (36 weeks in 2007, 52 weeks in 2008 and 16 weeks in 2009) as shown in the last column of the above table. For more details see section Data in the text.

Note that the data in Table 1 is data aggregated by week (for 104 weeks) for all (panellist) consumers. So, Table 1 gives the summary statistics for all variables used in our aggregate analysis, where the variables of interest are aggregated at the week level and by major sustainable product groups. This compactification makes the analysis of the trajectories of *aggregate* expenditures on sustainable goods tractable by focussing on trends at the weekly level.

## 3 Results

### 3.1 Exploratory graphical analysis

We begin our empirical analysis by looking at simple scatter plots of the expenditure shares of various eco-labelled products over time. Note that although we do not have an exact date for the start of the recession, week 65 in our data corresponds to the week when the UK government announced a £37 billion rescue package for Royal Bank of Scotland (RBS), Lloyds TSB and HBOS. This was on 13 October, 2008. We consider this week as the week of the onset of the recession. A dashed vertical line in our diagrams marks week 65. We also note that a solid vertical line in our diagrams marks week 46, which is the date at which the supermarket chain began applying carbon labels of its own on the first of several products.

**Fig 1:**
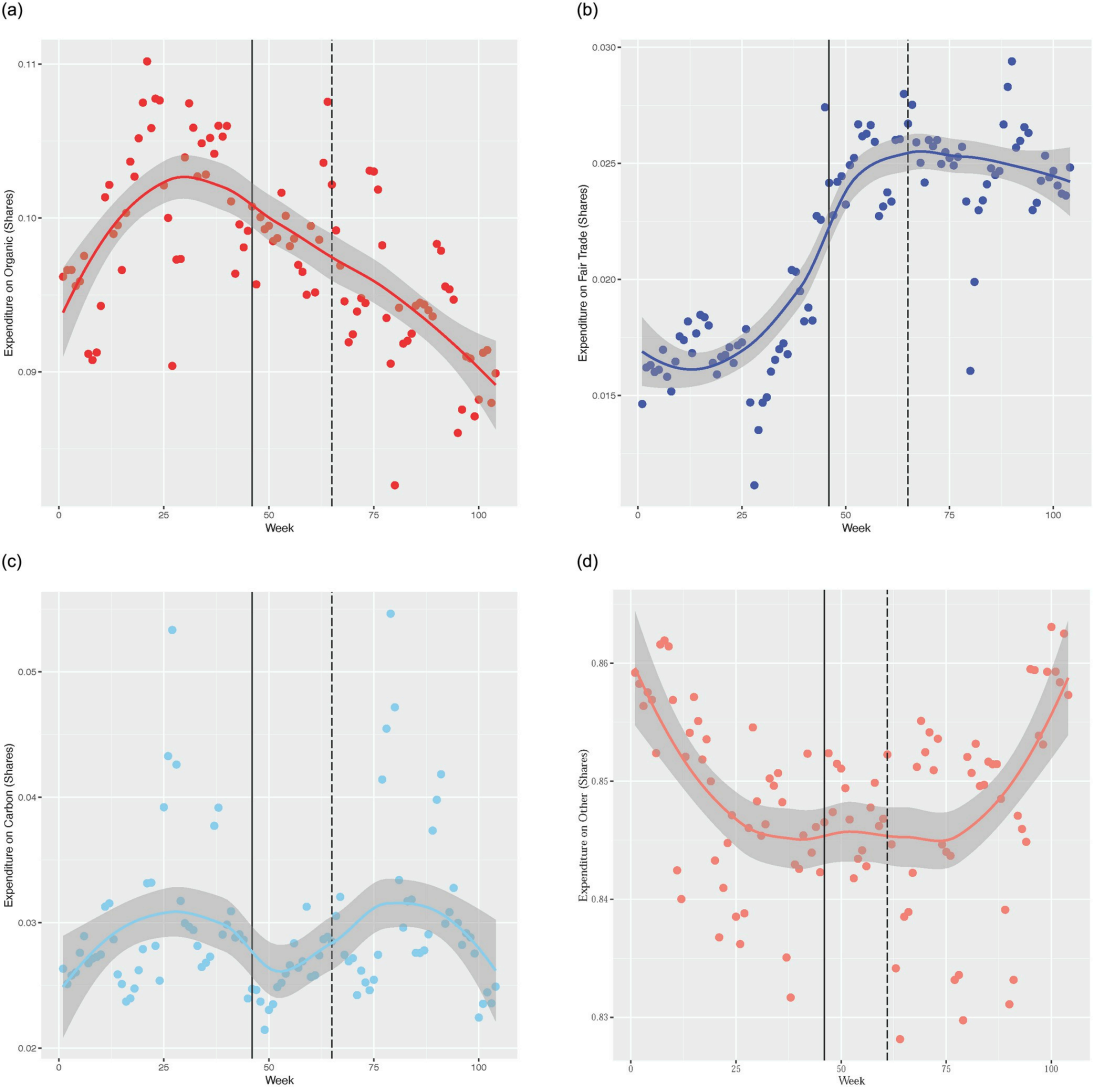
Shows the loess fit on expenditure shares of various eco-labelled product categories on the vertical axis over time in weeks on the horizontal axis. A dashed vertical line in our diagrams marks week 65. We consider this week as the week of the onset of the recession. A solid vertical line in our diagrams marks week 46 which is the week in which the supermarket chain began applying carbon labels on the first of several products. (a) Organic expenditure shares. (b) Fair trade expenditure shares. (c) Carbon expenditure shares. (d) Other expenditure shares.

As the scatter plots of these four aggregate product categories show a considerable degree of volatility and it is difficult to say without any smoothing of the data what the general trend is for each of these eco-labelled product categories. We use simple smoothers to examine the trends in the expenditure shares of these product categories. A natural starting point for smoothing data is the lowess smoother. Essentially a lowess smoother tries to fit a “local” linear regression to a set of points *y*_*t*_ around the point of interest *y* in the data where *y*_*t*_ is sufficiently close to *y* (the closeness is dictated by a selected bandwidth). As these methods are well known we do not outline details of the method. We refer the reader to [34] for further details. Sub-figures 1a-1d in Fig 1 show a loess fit to the data using default band width settings. We use a data driven cross-validation method to select the bandwidth when we model the relationship between expenditure shares of eco-labelled products and time using kernel based methods. The loess smoothers clearly show that organic expenditure shares fall over time (Fig 1a). Fair trade shares seems to rise over time (Fig 1b). For categories carbon and other, we see almost a flat trend line (Fig 1c and 1d). These results are roughly in line with our conjecture that the sales of fair trade products have held up and that the sales of organic products have plummeted during the recession. Sales of carbon and the category “other”, do not seem to have changed very much over the same time period. We note here that the carbon label for products came into existence as late as week 46 of our data. So the category carbon comprises of products that were labelled after the beginning of our sample period. Thus the carbon labelled category comprises of products that are “retrospectively” assigned as carbon labelled from week 1. This does not create any problem in our analysis. We see exactly the same flat trend in sales pre and post week 46. It is clear that the share of consumer expenditure on carbon labelled products has not fallen or risen during the recession. Carbon shares are fairly constant but a slight cyclicity is visible in the trend.

**Fig 2:**
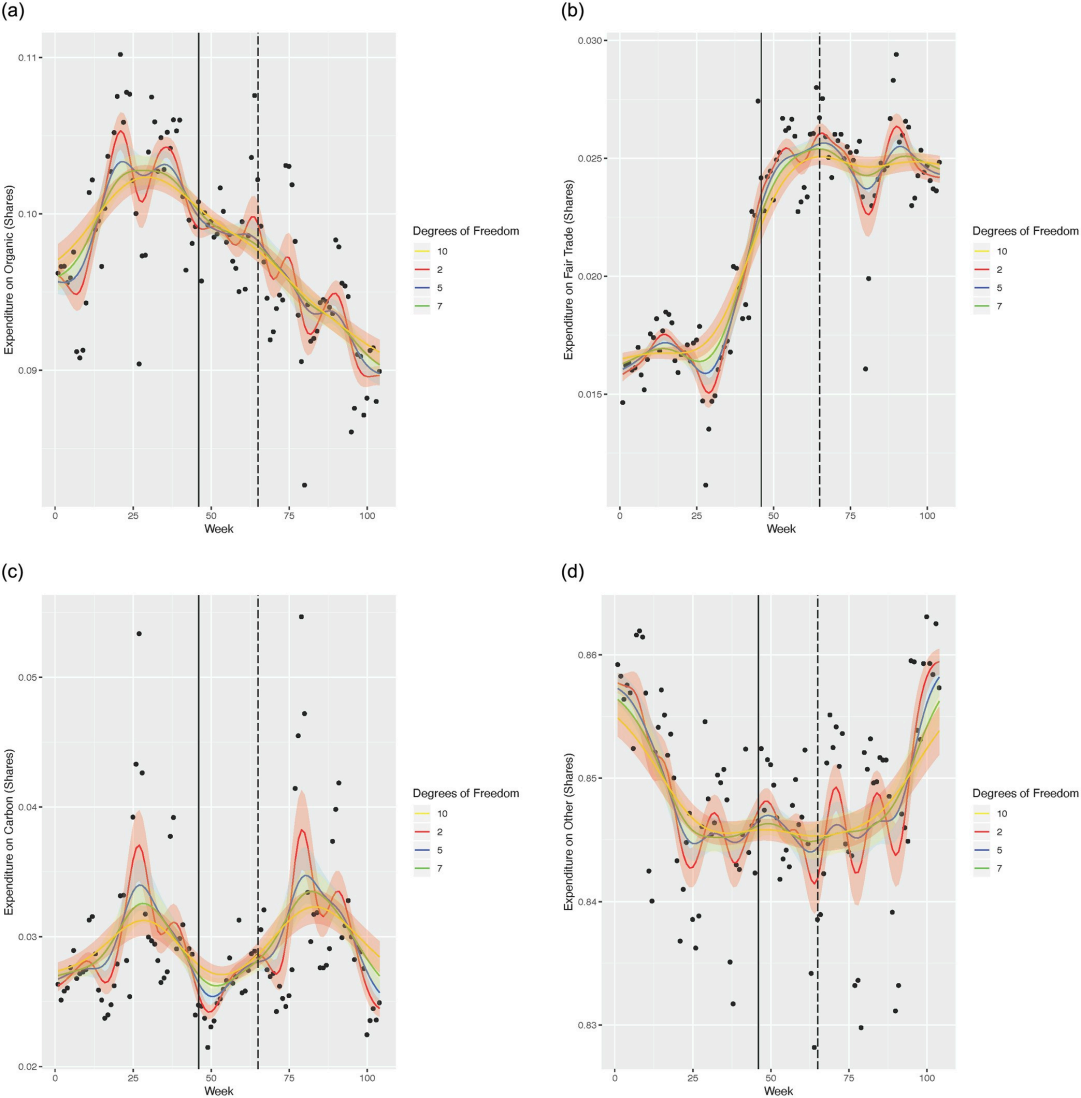
Shows the non-parametric fit on expenditure shares of various eco-labelled product categories on the vertical axis over time in weeks in the horizontal axis. A dashed vertical line in our diagrams marks week 65. We consider this week as the week of the onset of the recession. A solid vertical line in our diagrams marks week 46 which is the week in which the supermarket chain began applying carbon labels on the first of several products. (a) Organic expenditure shares. (b) Fair trade expenditure shares. (c) Carbon expenditure shares. (d) Other expenditure shares.

For robustness we also fit a non-parametric kernel based smoother to our data. We use the non-parametric kernel-based model specification test outlined in [35] which tests for consistent model specification. We use the command npcmstest in the package np in R to implement this test. In all cases, whether for the linear specification or for a flexible parametric specification involving squared terms and higher powers of the independent variable, this test strongly rejects the null hypothesis of the (parametric) regression specification in favour of a non-parametric specification (*p*-value <0.000). For our kernel based smoother, we use the well known Gaussian kernel as our kernel. The Gaussian kernel is given by $N(0,\sqrt{h})$ where *h* is the window width or bandwidth. For details see [34]. Sub-figures 2a-2d in Fig 2 show the results of this exercise. We use default span width settings for this graph. The results in Fig 2 convincingly establish the trends noted in earlier figures. We see organic expenditure shares fall over time (Fig 2a). Fair trade shares seems to rise over time (Fig 2b). For categories carbon and other, we see a flat trend line (Fig 2c and 2d). These results are roughly in line with our conjecture that the sales of fair trade products have held up and that the sales of organic products have plummeted during the recession. Sales of carbon and the category “other”, do not seem to have changed very much over the same time period. We note here that the carbon label for products came into existence as late as week 46 of our data. So the category carbon comprises of products that were labelled after the beginning of our sample period. Thus the carbon labelled category comprises of products that are “retrospectively” assigned as carbon labelled from week 1. This does not create any problem in our analysis. We see exactly the same flat trend in sales pre and post week 46. It is clear that the share of consumer expenditure on carbon labelled products has not fallen or risen during the recession. Carbon shares are fairly constant but a slight cyclicity is visible in the trend.

To sum up, our results from the graphical analysis at the aggregate level seem to clearly suggest that organic expenditure shares fall over time and a clear dip in the trajectory is visible, post week 65. For fair trade expenditure shares exactly the opposite trend is observed—for fair trade products we observe a rising trend in expenditure shares and post week 65, the trend is quite steep. For the carbon labelled category no clear trends is discernable except for a slight cyclicity over the period considered. The category “other” also shows a quite flat trend over most of the data period.

### 3.2 Econometric model for disaggregated purchases

In the previous section we have looked at *aggregate* (over all consumers) expenditure trends for eco-labelled products in the data. We now consider a very simple econometric model of purchase behaviour at the *disaggregated* or the individual consumer level to examine changes in the expenditures of the different eco-labelled varieties pre and post recession. This is the data in S2 File. Disaggregated consumer level expenditure data.

To model the purchase behaviour of individuals, let us consider a number (at least two) of time periods indexed *t* = 1, 2, …, *T*. Let us consider a number (at least two) of customers indexed *k* = 1, 2, …, *N*. Let *y*_*kt*_ be the spend on a category of items (organic or fairtrade) by customer *k* in period *t*. Consider the following model of consumer purchases:

$$\begin{array}{c} y_{kt}=\delta_{t}+\alpha_{k}+\gamma D_{t}+\beta s_{kt}+\lambda X_{\text{kt}}+u_{kt} \end{array}$$ (1)

where *α*_*k*_ is the consumer fixed effect and *δ*_*t*_ is a time fixed effect. We note that the term *δ*_*t*_ can encompass availability effects. *D*_*t*_ is a crunch dummy, i.e., it is 0 pre-crunch and is 1 post-crunch. *s*_*kt*_ denotes the total spend of customer *k* in period *t*. **X**_**kt**_ denotes a vector of controls and *u*_*kt*_ are the disturbance terms.

Taking differences over time in Eq 1 gives us:

$$\begin{array}{c} {△}_{t}y_{kt}={△}_{t}\delta_{t}+\gamma {△}_{t}D_{t}+\beta {△}_{t}s_{kt}+\lambda {△}_{t}X_{\text{kt}}+{△}_{t}u_{kt} \end{array}$$ (2)

Note that the above equation in differences eliminates unobserved customer fixed effects. Now to simplify the above, let us consider only two time periods—pre- and post-crunch. Then we get:

$$\begin{array}{c} △y_{k}=\delta +\gamma +\beta △s_{k}+\lambda △X_{k}+△u_{k} \end{array}$$ (3)

where the △ symbol indicates differences across the two periods and *δ* = △*δ*_*t*_. We assume for our analysis that the time trends for spends for a specific category of eco-labeled product is the same over the two periods being compared. This is an identifying assumption. We think that the assumption of similar trends is justified. The UK economy is characterised by political stability and low inflation. We are also not aware of any other policies or sectoral trends around the period of our analysis that might invalidate this assumption. Note that we can not identify *δ* or *γ* without additional restrictions. We can conceive of a more complicated model here that allows for differential responses to the crunch and differential responses to total spend. Formally, this differential response can be modelled by having a term *γ*_*k*_ and a term *β*_*k*_ for individual *k* in specification 1. We could then think of the term *γ*_*k*_ as comprised of *γ*_*k*_ = *γz*_*k*_ and *β*_*k*_ = *β* + *ηz*_*k*_ where *z*_*k*_ is some observable characteristic of *k*. To make this approach tractable, we could further posit *z*_*k*_ = *y*_*k*0_ the value of *y*_*kt*_ in the initial period. The use of *y*_*k*0_ reflects the idea that those who buy high levels of the product group may react differently to changes in total spend.

To operationalise Eq 3 for our data, we divide our individual level or disaggregated data into four periods. We divide our data into periods as follows: 1. weeks 1 to 12 (period 0), 2. weeks 13 to 52 (period 1), 3. weeks 53 to 64 (period 2) and 4. weeks 65 to 104 (period 3). Recall that we posit that the crunch started from week 65 in our data. For our analysis, we use two periods, the post-crunch (period 3 from weeks 65 to 104) and a matching pre-crunch period (period 1 from weeks 13 to 52). Note this leaves two other periods, namely weeks 1 to 12 and weeks 53 to 64 that we do not use in our analysis. We are reluctant to exploit the second of these periods as there is some uncertainty on when to date the start of the crunch. A recession affects an economy over time. Recessionary effects do not arrive at a well-defined point in time. Our regression specification also takes into account the fact that recessionary effects do not arrive at a well-defined point in time. For this reason we allow for a gap (weeks 53 to 64) to allow recessionary effects to have an effect on consumer purchases. With reference to these periods used in our regression specification, we can be reasonably sure that the recessionary effects are unlikely to start before period 1 and are surely in effect during period 3. So, while the precise start of recessionary trends is hard to identify, our approach obviates the need to identify a specific start date of the onset of the recession. We also note that while recessionary effects also play out through other channels, for example, unemployment, we are concerned only with the impact of the recession on the purchases of eco-labelled products. The other period (weeks 1 to 12) has to be discarded since we need periods spanning equal number of weeks (like period 1 and period 3) to make meaningful comparisons of possible changes in the magnitude of expenditure shares.

Traditional price theory predicts that the effect of the economic recession on all types of eco-labelled products should be the same (and that consumer taste remains unchanged regardless of economic conditions). That is, the negative economic shock of the recession should cause a reduction in consumer spending during an economic downturn as consumers become more price aware. With this in mind we operationalize a set of control variables in the vector **X**_**k**_. Controls in the vector **X**_**k**_ are dictated by traditional price theory. We assume as in traditional price theory (see [30]) that consumer preferences are unchanged during an economic recession. We control for price (please see details in the next paragraph of how price indices are calculated) and various promotions like discounts, markdown, etc. which may affect sales, demographic characteristics of consumers that may affect sales and changes in total expenditure which proxies for changes in income (or the total consumer spend).

We use our earlier classification of the 4 types of eco-labelled products and generate a few summaries from our disaggregated data for each of the 4 periods. For each customer *k* = 1, …, *N*, each eco-labelled group *g* = 1, 2, 3, 4 and each period *t* = 0, 1, 2, 3 combination, we generate summaries of our data aggregated at the consumer level (not at the week level as we had done earlier). These summaries include the total spend by the customer *k* on items in group *g* in period *t*, the total number of transactions by the customer *k* on items in group *g* in period *t*, the number of transactions by the customer *k* on items in group *g* in period *t* in which a promotion was redeemed, the number of transactions by the customer *k* on items in group *g* in period *t* in which the product was marked down, the number of transactions by the customer *k* on items in group *g* in period *t* in which the price was discounted. So, for example, for each consumer *k* we generate summaries that give us the sum of weekly spends on organic items for period *t* = 0, 1, 2, 3, the sum of weekly transactions on organic items for period *t* = 0, 1, 2, 3, sum of weekly organic transactions bought under promotion for period *t* = 0, 1, 2, 3, sum of weekly organic transactions bought under mark down for period *t* = 0, 1, 2, 3, sum of weekly organic transactions bought under price discount for period *t* = 0, 1, 2, 3. We create similar sequences of 24 fields as organic for the other eco-labelled categories like fairtrade, carbon and other. We also create measures of price for each consumer *k* for each product group *g* for period *t* = 0, 1, 2, 3. The way in which the price is calculated for the product group *g* for each customer *k* is the following. We calculate the average price for each transaction by the customer *k* on items in group *g* in period *t* where quantity is positive. We then add up these average prices and at the end of the run we divide this number by the number of transactions of the consumer for that product group *g*. We note that our (simple) price indices are not quantity weighted. We generate measures of quantity for each customer *k* for each product group *g* by dividing the consumer spend by the measure of price. We also have the percentage of all spends for each eco-labelled group *g* bought under promotion, markdown or discount by customer *k*, the sum of weekly spends for period *t* = 0, 1, 2, 3, the sum of weekly items bought by customer *k* for period *t* = 0, 1, 2, 3, sum of weekly visits by customer *k* for period *t* = 0, 1, 2, 3 and the sum of weekly items bought by customer *k* under promotion for period *t* = 0, 1, 2, 3 and the number of “green points” awarded to customer *k*.

For the variables included in our regression specification, we take the natural logarithm of all of the aforementioned variables and then take the difference across periods 3 and 1. So, we have the difference in organic spends over two periods, the difference in fair trade spends across two periods, and so on. We also generate the difference in the percentage of all goods bought under promotion, markdown or discount under each labelled group, such as organic, fair trade, and so on. Table 2 shows the summary statistics for the main variables of these statistics (at the disaggregated consumer level).

**Table 2:** Disaggregated data at consumer level.

Variable	Mean	Std. Dev.	Min.	Max.	N
Change in organic spending	-0.195	0.894	-5.063	5.46	59390
Change in organic products promotion	0.039	0.275	-2.485	3.332	59390
Change in organic products markdown	0.006	0.291	-2.639	3.091	59390
Change in organic products discount	0.198	0.527	-2.303	4.111	59390
Change in organic prices	0.049	0.411	-3.912	4.564	59390
Change in fairtrade spending	0.069	0.487	-3.298	4.926	59390
Change in fairtrade products promotion	0	0.066	-1.946	2.079	59390
Change in fairtrade products markdown	0.002	0.05	-1.386	1.946	59390
Change in fairtrade products discount	0.01	0.109	-1.099	2.398	59390
Change in fairtrade prices	0.01	0.264	-3.912	3.127	59390
Change in carbon spending	-0.045	0.904	-5.16	4.743	59390
Change in carbon products promotion	0.011	0.506	-3.689	4.025	59390
Change in carbon products markdown	-0.002	0.069	-2.303	1.386	59390
Change in carbon products discount	0.062	0.279	-2.485	2.944	59390
Change in carbon prices	0.021	0.38	-2.582	3.912	59390
Change in spending (pre vs. post recession)	-0.171	0.963	-8.866	4.868	59390
Older Adults (Stage 1)	0.106	0.308	0	1	59390
Older Families (Stage 2)	0.193	0.395	0	1	59390
Pensioners (Stage 3)	0.019	0.137	0	1	59390
Young Adults (Stage 4)	0.184	0.387	0	1	59390
Young Families (Stage 5)	0.322	0.467	0	1	59390
Other (Stage 6)	0.176	0.381	0	1	59390
Green points for consumer	4.734	2.023	0	12.338	59390

**Note**: The above table shows summary statistics for consumer level expenditure and transactions information on each major eco-labelled product category—organic, fair trade, carbon and the category, “other”. Figures show changes in the log value of variables over two periods—pre-recession (weeks 13 to 52, or period 1) and post recession (weeks 65 to 104, or period3). These statistics are compiled from scanner-level (revealed preference) data on food consumption recorded at a leading UK retailer with a market share over 31 percent in 2008. Our empirical analysis focuses on weekly observations for financial week 17 of 2007 up to and including financial week 15 in 2009. Thus the date covers a period of 104 weeks (36 weeks in 2007, 52 weeks in 2008 and 16 weeks in 2009) as shown in the last column of the above table. For more details see section Econometric model for disaggregated purchases in the text.

Next, following regression specification 3, we regress the difference in organic spending over the two periods (period 1 and period 3) on a constant which gives us the mean difference in consumer spending on organic products over the two periods. Recall that the regression specification 3 is the following:

$$\begin{array}{c} △y_{k}=\delta +\gamma +\beta △s_{k}+\lambda △X_{k}+△u_{k} \end{array}$$

where the △ symbol indicates differences across the two periods and *δ* = △*δ*_*t*_. We include additional controls in the regression specification such as the change in organic prices over the two periods, the change in the aggregate percentage of organic goods bought under promotion, markdown or discount, over the two periods. We also include some demographic descriptors corresponding to the life stage of the club card account holder, as mentioned in section Data, as well as the number of “green points” a club card account has earned. We repeat this exercise for other eco-labelled categories like fair trade, etc. and for the category “other”. Our regression results (for specification 3) is reported under Tables 3–6.

**Table 3:** Effect of the recession on organic products^a^^b^.

	1	2	3	4	5	6
**Constant**	-0.195***	-0.198***	-0.165***	-0.262***	-0.271***	-0.086***
	(0.004)	(0.004)	(0.004)	(0.004)	(0.010)	(0.013)
Change in organic prices		0.068***	0.063***	0.105***	0.106***	0.109***
		(0.009)	(0.009)	(0.008)	(0.008)	(0.008)
Change in spending (pre vs. post recession)			0.191***	0.155***	0.155***	0.165***
			(0.004)	(0.004)	(0.004)	(0.004)
Change in organic products promotion				0.137***	0.139***	0.146***
				(0.013)	(0.013)	(0.013)
Change in organic products markdown				0.638***	0.638***	0.636***
				(0.012)	(0.012)	(0.012)
Change in organic products discount				0.399***	0.401***	0.419***
				(0.007)	(0.007)	(0.007)
Older Adults (Stage 1)					0.000	0.000
					(.)	(.)
Older Families (Stage 2)					0.058***	0.046***
					(0.013)	(0.013)
Pensioners (Stage 3)					-0.018	-0.008
					(0.026)	(0.026)
Young Adults (Stage 4)					0.047***	0.023^+^
					(0.013)	(0.013)
Young Families (Stage 5)					-0.031**	-0.037**
					(0.012)	(0.012)
Other (Stage 6)					-0.004	-0.019
					(0.013)	(0.013)
Green points for consumer						-0.037***
						(0.002)
No. of Obvs.	59390	59390	59390	59390	59390	59390

^a^ For each column the dependent variable is the change in log organic spending over two periods—pre-recession (weeks 13 to 52, or period 1) and post recession (weeks 65 to 104, or period 3). Column (1) shows the dependent variable regressed on a constant term following specification 3 in the text. So, the coefficient reported as constant in the above table shows the average change in log organic spending over the two periods. This is the percentage change in organic spending over the two periods. The rest of the columns modify the specification in 3 by including additional control variables which are specified in the rows of the first column of the table.

^b^ **Note**: *t*-statistics reported under each coefficient in parenthesis. Significance at:

^+^*p* < 0.10

*p < 0.05,

**p < 0.01,

***p < 0.001.

**Table 4:** Effect of the recession on fairtrade products^a^^b^.

	1	2	3	4	5	6
**Constant**	0.069***	0.066***	0.073***	0.059***	0.059***	0.031***
	(0.002)	(0.002)	(0.002)	(0.002)	(0.006)	(0.007)
Change in fairtrade prices		0.252***	0.246***	0.258***	0.258***	0.258***
		(0.008)	(0.007)	(0.007)	(0.007)	(0.007)
Change in spending (pre vs. post recession)			0.039***	0.034***	0.034***	0.033***
			(0.002)	(0.002)	(0.002)	(0.002)
Change in fairtrade products promotion				1.267***	1.267***	1.267***
				(0.028)	(0.028)	(0.028)
Change in fairtrade products markdown				1.265***	1.264***	1.260***
				(0.038)	(0.038)	(0.038)
Change in fairtrade products discount				1.004***	1.003***	0.998***
				(0.017)	(0.017)	(0.017)
Older Adults (Stage 1)					0.000	0.000
					(.)	(.)
Older Families (Stage 2)					-0.007	-0.005
					(0.007)	(0.007)
Pensioners (Stage 3)					0.048***	0.047**
					(0.015)	(0.015)
Young Adults (Stage 4)					-0.005	-0.001
					(0.007)	(0.007)
Young Families (Stage 5)					0.003	0.003
					(0.007)	(0.007)
Other (Stage 6)					0.002	0.004
					(0.007)	(0.007)
Green points for consumer						0.006***
						(0.001)
No. of Obvs.	59390	59390	59390	59390	59390	59390

^a^ For each column the dependent variable is the change in log fairtrade spending over two periods—pre-recession (weeks 13 to 52, or period 1) and post recession (weeks 65 to 104, or period 3). Column (1) shows the dependent variable regressed on a constant term following specification 3 in the text. So, the coefficient reported as constant in the above table shows the average change in log fairtrade spending over the two periods. This is the percentage change in fairtrade spending over the two periods. The rest of the columns modify the specification in 3 by including additional control variables which are specified in the rows of the first column of the table.

^b^ **Note**: *t*-statistics reported under each coefficient in parenthesis. Significance at:

^+^*p* < 0.10

*p < 0.05,

**p < 0.01,

***p < 0.001.

**Table 5:** Effect of the recession on carbon products^a^^b^.

	1	2	3	4	5	6
**Constant**	-0.045***	-0.050***	-0.025***	-0.093***	-0.096***	-0.010
	(0.004)	(0.004)	(0.004)	(0.003)	(0.009)	(0.012)
Change in carbon prices		0.257***	0.235***	0.254***	0.254***	0.255***
		(0.010)	(0.010)	(0.008)	(0.008)	(0.008)
Change in spending (pre vs. post recession)			0.147***	0.093***	0.093***	0.097***
			(0.004)	(0.003)	(0.003)	(0.003)
Change in carbon products promotion				0.920***	0.920***	0.921***
				(0.006)	(0.006)	(0.006)
Change in carbon products markdown				0.993***	0.993***	0.986***
				(0.043)	(0.043)	(0.043)
Change in carbon products discount				0.803***	0.805***	0.815***
				(0.011)	(0.011)	(0.011)
Older Adults (Stage 1)					0.000	0.000
					(.)	(.)
Older Families (Stage 2)					-0.005	-0.011
					(0.011)	(0.011)
Pensioners (Stage 3)					-0.046*	-0.041^+^
					(0.023)	(0.023)
Young Adults (Stage 4)					0.022+	0.012
					(0.011)	(0.011)
Young Families (Stage 5)					-0.004	-0.007
					(0.011)	(0.011)
Other (Stage 6)					0.015	0.008
					(0.012)	(0.012)
Green points for consumer						-0.017***
						(0.001)
No. of Obvs.	59390	59390	59390	59390	59390	59390

^a^ For each column the dependent variable is the change in log carbon spending over two periods—pre-recession (weeks 13 to 52, or period 1) and post recession (weeks 65 to 104, or period 3). Column (1) shows the dependent variable regressed on a constant term following specification 3 in the text. So, the coefficient reported as constant in the above table shows the average change in log carbon spending over the two periods. This is the percentage change in log carbon spending over the two periods. The rest of the columns modify the specification in 3 by including additional control variables which are specified in the rows of the first column of the table.

^b^ **Note**: *t*-statistics reported under each coefficient in parenthesis. Significance at:

^+^*p* < 0.10

*p < 0.05,

**p < 0.01,

***p < 0.001.

**Table 6:** Effect of the recession on other products^a^^b^.

	1	2	3	4	5	6
**Constant**	-0.230***	-0.234***	-0.093***	-0.321***	-0.321***	-0.281***
	(0.003)	(0.004)	(0.002)	(0.003)	(0.006)	(0.007)
Change in other prices		0.056**	-0.212***	-0.145***	-0.145***	-0.145***
		(0.020)	(0.012)	(0.010)	(0.010)	(0.010)
Change in spending (pre vs. post recession)			0.721***	0.596***	0.596***	0.597***
			(0.002)	(0.002)	(0.002)	(0.002)
Change in other products promotion				0.112***	0.112***	0.112***
				(0.002)	(0.002)	(0.002)
Change in other products markdown				0.054***	0.054***	0.054***
				(0.003)	(0.003)	(0.003)
Change in other products discount				0.228***	0.228***	0.231***
				(0.002)	(0.002)	(0.002)
Older Adults (Stage 1)					0.000	0.000
					(.)	(.)
Older Families (Stage 2)					0.001	-0.001
					(0.007)	(0.007)
Pensioners (Stage 3)					0.005	0.007
					(0.014)	(0.014)
Young Adults (Stage 4)					0.003	-0.002
					(0.007)	(0.007)
Young Families (Stage 5)					-0.003	-0.004
					(0.006)	(0.006)
Other (Stage 6)					-0.004	-0.007
					(0.007)	(0.007)
Green points for consumer						-0.008***
						(0.001)
No. of Obvs.	59390	59390	59390	59390	59390	59390

^a^ For each column the dependent variable is the change in log other spending (where other refers to periods—pre-recession (weeks 13 to 52, or period 1) and post recession (weeks 65 to 104, or period 3). Column (1) shows the dependent variable regressed on a constant term following specification 3 in the text. So, the coefficient reported as constant in the above table shows the average change in log other spending over the two periods. This is the percentage change in other spending over the two periods. The rest of the columns modify the specification in 3 by including additional control variables which are specified in the rows of the first column of the table.

^b^ **Note**: *t*-statistics reported under each coefficient in parenthesis. Significance at:

^+^*p* < 0.10

*p < 0.05,

**p < 0.01,

***p < 0.001.

Tables 3–6 confirm the intuition that we had earlier by looking at the aggregate trends in the graphs of the weekly expenditure shares of eco-labelled products.

From the results in Table 3 we see that there is a fall in organic spending of almost 19% (recall that this number denotes the percentage change in quantity over the initial (pre-recession or period 1) and the final (post-recession or period 3) periods). The positive signs on the changes in promotions, markdown and discounts is not surprising. From the coefficient of the life stage variables, it seems that compared to older adults (the base category shown with a coefficient of 0 in the regression table) pensioners and young families fared poorly, which is what we would expect. Young adults seem to do better than older adults and so do older families. We interpret the negative coefficient of green points as indicative of a negative relation between the number of “green points” accumulated (perhaps indicating a more environmentally conscious consumption pattern) and the fall in organic spending (reinforcing the main effect of the fall in organic spend).

In Table 4 we see a mildly positive increase in fair trade spending over the two periods. Interestingly, pensioners spend more than older adults (base category) on fair trade. Also now the sign on “green points” is positive indicating that more environmentally friendly consumers are more likely on average to have higher spend on fair trade products.

Tables 5 and 6 show expected results. Overall on average expenditure shares on both carbon labelled products and the residual category “other” decline over the two periods.

So with the exception of fair trade products none of the other eco-labelled product categories are able to sustain sales during the recession.

We also note that in Tables 3–6 the sign on the coefficient denoting the change in spending for the various eco-labelled categories (shown in bold) is robust across the multiple specifications considered.

## 4 Alternative underpinnings of eco-labelled consumption

In this section, we review the store availability of eco-labelled products and look at the trajectory of prices by eco-label category, during the period covered by our data. We do this to exclude shelf space allocation and divergent product prices as potential confounding factors in our analysis. Next, we discuss the traditional economic perspective on the impact of a recession on consumer purchases and show that this traditional perspective cannot explain our empirical results. We then turn to alternative explanations of our main findings including an exploration of consumers’ behavioural motives. We discuss how models of context-dependent behaviour, specifically salience theory, can explain our observations. We also explore how identity considerations or personal norms and social image might affect individual choices in the context of eco-labelled food.

### 4.1 Supply availability

A potential confounder in our analysis could be the supply availability of eco-labelled products in stores. Retailers may cut back on specific eco-labelled ranges and shelf space and/or also promote other ranges. Such actions on the part of retailers could lead to endogenous changes in availability and could affect the expenditure shares of eco-labelled products.

We note that from the early 2000s, the market for fair trade products in the UK has been characterised by the main streaming of food products through conventional retail outlets, particularly supermarket multiples. Supermarket multiples is a defined sub-set of the major supermarkets, the major ones are: Tesco, Sainsbury, Asda, Morrison, Co-operative and Waitrose. It excludes discount retailers (Aldi, Lidl). As part of this development, the said supermarket chain launched its own brand fair trade line in March 2004. In its 2004/5 annual report on corporate sustainability the supermarket chain highlighted that it was stocking 90 food products including 14 own brand products. By 2006/7 the number increased to 130 fair trade lines of which 30 were the supermarket chain’s own-label products and in 2007/2008, UK stores of the said supermarket chain carried 188 fair trade products including 117 fair trade labelled own-brand products (The Institute of Grocery Distribution, 2015).

In January 2007, the management of this supermarket chain announced a switch of attention to carbon labelling and this label was gradually introduced on its own brand products in the UK. The annual report on corporate sustainability in 2009 and later years no longer mentions fair trade. In short: we feel that supply availability cannot be held responsible for the patterns in expenditure shares of fair trade products that we observe for 2008 and for the first months of 2009. Unfortunately, in our sample we do not have information on the availability of a product over all stores to account for availability.

### 4.2 Price changes

UK households experienced a negative shock to their income during the recession. The negative shock was further exacerbated by an increase in the real price of food which has remained high ever since. Food prices peaked in 2008, when the annual rate of food price inflation was 5.5 percent. Although food prices started to fall in February 2009, the average annual growth rate was still almost 3.8 percent between 2007 and 2009. This increase in the price of food was unevenly distributed; there were big changes in the relative prices of different food groups. The period 2008–2009 was characterized by a high degree of volatility and the price changes did not occur at the same time across different goods [36].

**Fig 3:**
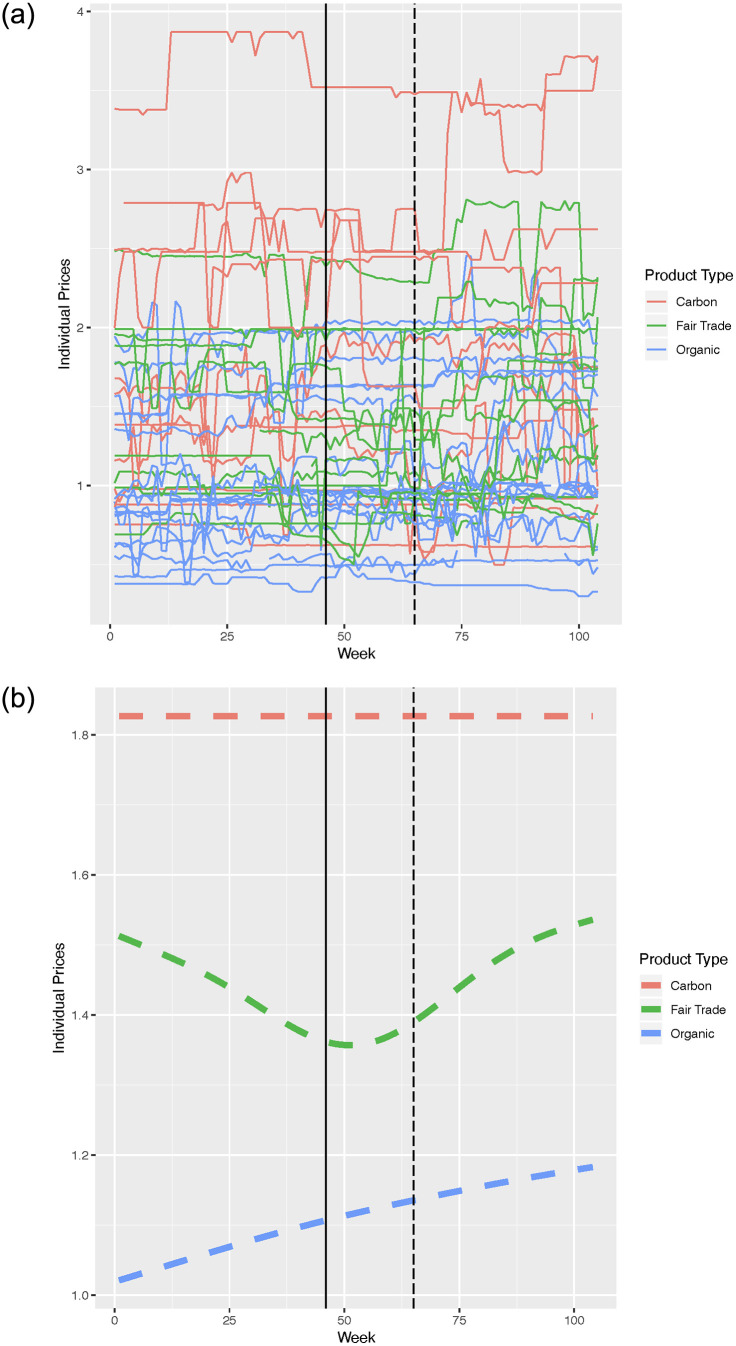
(a) Above shows price trajectories for individual products belonging to different eco-labelled categories for 104 weeks. The products are grouped by category into 3 different eco-labelled product groups as shown in the legend alongside. Price is plotted on the vertical axis against time in weeks in the horizontal axis. (b) Shows a price index constructed as an average of individual prices. A dashed vertical line in our diagrams marks week 65. Week 65 in our data can be regarded as the date of the onset of the recession. A solid vertical line in our diagrams marks week 46 which is the week in which the supermarket chain began applying carbon labels on the first of several products.

In this section we construct simple price indices to study price trajectories for the different eco-labelled categories used in our analysis. We use the aggregated data (aggregated at the week level) for this analysis. Recall that in our sample we have information on expenditure and quantities bought for individual products for 104 weeks. These products have also been classified as belonging to one of the following three main eco-labelled groups: organic, fair trade and carbon. From the information on expenditure and quantities purchased for individual products, we back out prices for individual products. To obtain these prices we divide the expenditure on individual products by the quantities purchased of these products. We obtain these prices for individual products for each week in our sample. We plot the price trajectories of these individual products over the 104 weeks in our sample. We show these individual level price trajectories in sub figure 3a of Fig 3.

The individual price trajectories are too noisy to lead to any meaningful conclusion regarding the price trends for any of the three aforementioned categories of products. To get a better sense of the price trends, we create a simple price index for each of the three categories of products. We aggregate the prices of the individual products by week for each of the three categories—organic, fair trade and carbon. So for each week we calculate a simple average of the individual prices of products by eco-labelled category. This process then gives us three price indices, one for each category. Sub figure 3b in Fig 3 shows a plot of these price indices over time. Fig 3b shows that the price index for fair trade products was higher than the price index for organic products at the start of the 104 weeks investigated and remained higher throughout. So fair trade was more expensive than organic throughout our sample period. Fig 3b also shows that the category carbon was the most expensive during this period. These results suggest that the price index trajectory by category cannot explain the observed pattern in expenditure shares for fair trade products in 2008 and in the first quarter of 2009. In addition, the index for fair trade shows a stronger positive trend after week 50.

### 4.3 Theory of buyer behaviour

A recession affects consumer expenditures in (at least) two ways. First, a recession reduces disposable income and leads to a smaller budget available for consumption. Second, holding disposable income constant (e.g., for those households who are not affected financially), people tend to save more or pay down debts during a recession. This again leads to less money available to spend on goods and services (for an overview of how business cycle fluctuations affect consumer behaviour, from a marketing perspective, see the paper by [37]). The common assumption, in traditional economic analysis, is that a household’s taste does not change with changes in economic circumstances. Therefore, the utility a household derives from consumption at different levels of expenditure should be unaffected by the onset of a recession. Any adjustment in expenditure patterns seen during an economic recession would simply be due to changes in the consumption budget (see [31]). Following this logic, we assume that households trade-off the added utility of the more expensive eco-labelled variety of a food product against the utility of a standard food product, and in particular, the supermarket’s private label products (see for example, [38]). As the household gets poorer during a recession, the marginal utility of the standard product would rise relative to the marginal utility of the product with the eco-label. This would lead to a substitution toward the standard product. Under this assumption, a recession is expected to lead to smaller (observed) expenditure shares for the eco-labelled categories and larger (observed) shares for the standard category of food products. We would expect the same effect on the marginal propensity to consume regardless of whether the household’s financial loss is due to a loss of income or due to a parallel shift in prices, since money is treated as fungible in neoclassical economics [39]. To see the implications of our result for fungibility of income see **Note 4** in S1 Appendix.

The simple graphical analysis in section Results reveals that the trend we observe in our data is for the organic expenditure shares to fall and for fair trade shares to rise. The expenditure share for carbon-labelled food products shows little change. Regression analysis using disaggregated data confirms this result. Overall, the expenditure pattern observed in our data cannot be explained by the neoclassical income effect described above. First, indicators of U.K. households’ perception of their own financial situation showed a gloomy picture, in particular, from April 2008 to June 2009 (Office of National Statistics). The Eurobarometer Consumer survey asks respondents monthly how they think the general economic situation has changed over the last 12 months. A negative balance means respondents reported their financial situation got worse, a positive balance means they reported it improved. At its lowest, in May 2009, the Euro-barometer reported an aggregate balance of negative 82.3 for the general economic situation. In addition, food prices in the U.K. increased substantially during the early part of the recession, as mentioned earlier. This double squeeze of lower incomes and higher food prices put pressure on consumer expenditures.

### 4.4 Salience model

We now discuss some alternative explanations to account for our results. First, we discuss context dependent choice in which a consumer’s choice is drawn to salient attributes of a product, which in our case, is a product’s public good attribute (or lack of it).

The model of salience and consumer choice [18] combines two ideas. First, choices are made in context. Second, consumers evaluate products by comparing these products with other products they are thinking about purchasing. In this model, consumers focus on and thus overweigh product attributes that are salient. Salience is determined by the degree to which an attribute varies within an evoked set of options that are brought to mind by the purchase occasion. Thus, in this model, the context is determined by the choice set itself. Evidence suggests that consumers generally consider only a subset of the options available in the market. The typical number of options in such evoked sets ranges from two to five (see [40]). To see an example of how the salience model applies in our case please see **Note 1** in S1 Appendix.

In the salience framework it follows that attribute sensitivity depends on attribute levels; when all options in the set become more expensive the consumer will become less price sensitive/more quality sensitive. Another observation is that the addition of other options to the choice set has potential consequences for consumer choice because it affects the reference product and thus the attribute that stands out. Both observations have important implications in our case study. Note that in our case, the context would be a specific food category (e.g., dark chocolate bars or coffee) with goods varying in organic, fair trade or no eco-label, respectively. Consider a situation where the consumer is making a decision to buy coffee from the choice set indicated in the above set-up (so the consumer has fair trade and organic coffee choices to consider when making a decision). As shown by experimental evidence (for an overview see [41]), the salience perspective has stark implications for the effect of changes in price or consumer budgets. When only one specific good within a choice set is affected because of a price change (say free trade coffee only is affected), the salience model predicts that consumers will substitute to the lower public good quality of the good in this category (this means an increase in the share of cheaper organic coffee in total coffee sales). When in contrast, the change affects all goods in the category (all coffee) as in the case of an income change, the salience model predicts the consumer substitutes toward the higher public good quality good or free trade coffee in this case. To see a simple numerical example that makes this idea clear, please see **Note 2** in S1 Appendix.

The salience model leads to starkly different predictions for a price change and for an income change. For our scenario, where the recession led to a generic change in budgets, the salience model predicts that consumers became relatively less sensitive to price differences of products and instead focused on the public good quality of the product. One of our key empirical results—increasing fair trade shares and falling organic shares of products– is therefore clearly borne out by this model’s predictions.

### 4.5 A model of identity and social image

A second alternative theoretical explanation for our empirical results is offered by moral motivations and identity. Consumers who prefer to regard themselves as socially responsible individuals derive utility not only from the direct consumption of a good (the direct utility) but also from moral costs or rewards associated with the public good attribute of such consumption, i.e., identity. Following [32], identity is determined by a comparison of the actual consumption to the morally ideal consumption, the “right thing to do”. As explained by [33], this behaviour is socially-directed even though the focus here is on a personal moral norm. According to this economic model of moral motivation, individuals form beliefs about the moral standard through expectation about others’ behaviour. In our case, this would be the choice between eco-labelled food versus non-labelled food [42, 43].

A further theoretical explanation is offered by social image considerations. The influence of social norms in this context is through social distinction or reputation as a motivation for pro-social behaviour. [43] argue that social norms have an additional direct influence on behaviour parallel to personal norms but that—in the context of food consumption—the influence of social norms can be expected to be weak. Because food consumption is for the most part not a public activity, social reputation is considered (in general) less compelling in explaining food consumption than theories of identity or morality [44].

However, other recent studies suggest that for eco-labelled food and fair trade in particular, social norms do play a role [42]. Following [19, 20], one reason why consumers are willing to pay a price premium for an eco-labelled goods is that it generates a public good reputation for the buyer. When a consumer buys a public good attribute, which is incorporated in a traded good, this is, by construction, a signal of the consumer’s monetary valuation of the public good outcome. Thus the purchase of an eco-labelled good sends a clear signal of the buyer’s public good preferences. These choices in turn can lead to eco-labelled goods being purchased without the consumer questioning, or even considering, the effectiveness of the eco-label but merely to increase esteem [45]. Although purchasing an eco-labelled good in order to increase one’s own esteem is quite selfish, it can be an important factor in the demand for public good attributes. Indeed, [46] find this non-altruistic effect to be crucial in analysing the rise of the fair trade movement in particular. They argue that there would be no fair trade without this effect. [47] provide experimental evidence supporting self-signalling whereby consumers are partially motivated to buy a product for its public good attribute. Specifically they find crowding out of demand when price discounts dampen the self-signal of altruistic motivation.

**Fig 4:**
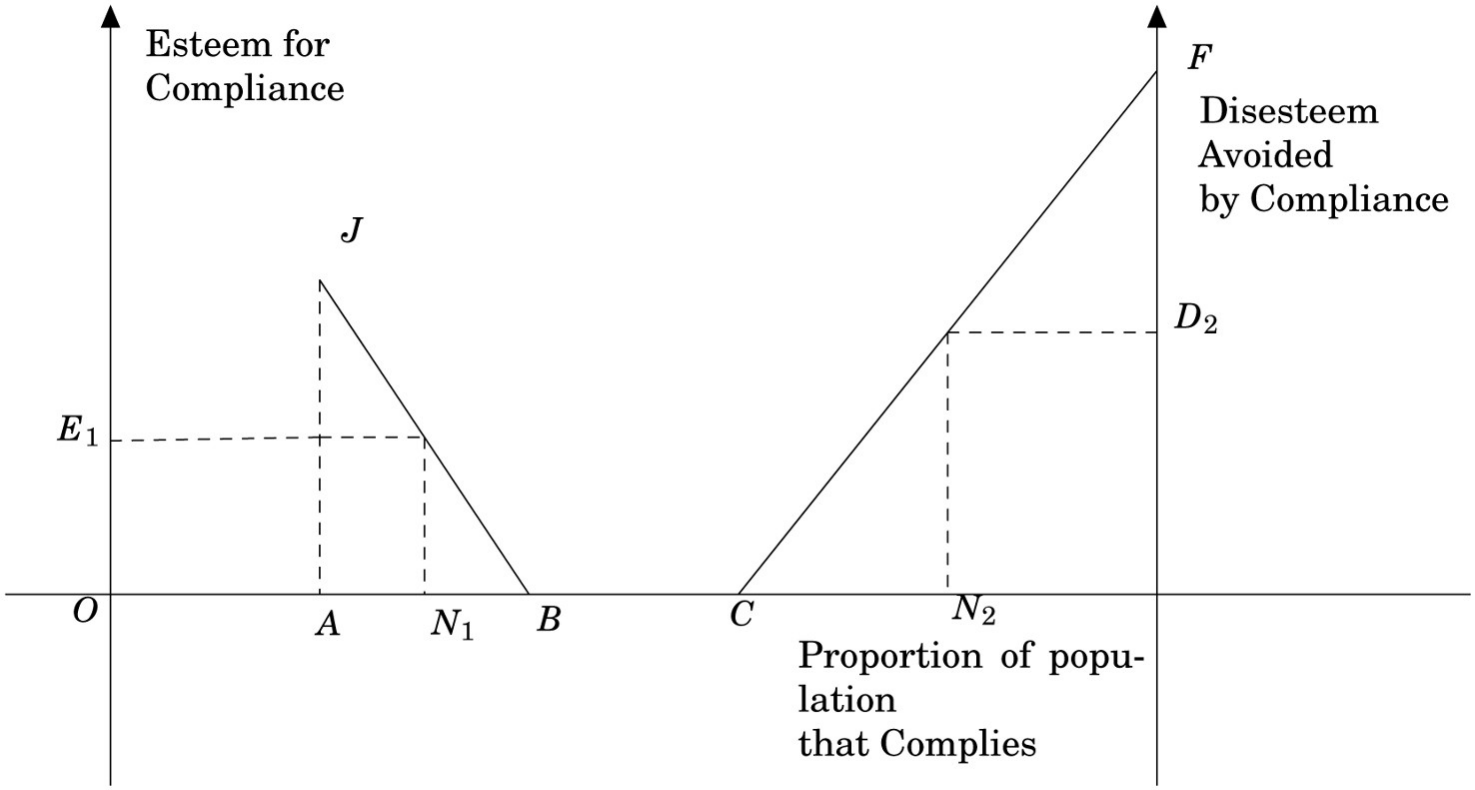
Shows the relation between esteem, disesteem and the proportion of a population that complies with a specific behavioural practice. Over the range 0 to *A*, the behaviour has not yet been registered as an ideal. Over the range *A* to *B* it becomes established. If the number of compliers is *N*_1_, an amount of esteem of *E*_1_ is allocated to all those who comply. As the proportion of the compliers increases from *A* to *B*, the esteem obtained from it decreases. At *B*, the behavioural practice has become so common that esteem is no longer is attached to it. In the final range from *C* to 100%, disesteem is attached to non-compliance whereas no esteem is available for compliance. If the number of compliers is *N*_2_, an amount of disesteem of *D*_2_ is allocated to all those who do not comply. In this range *C* to 100%, most comply with the behavioural practice and non-compliance is distinctive. The disesteem attached to non-compliance increases as the number of compliers increases. Source: [48], page 239.

One implication of the above results for our analysis is that the preference for buying the eco-labelled product is socially embedded. Esteem obtained is influenced by other consumers’ product choice and will change with the proportion of the population that is buying the product containing the pro-social characteristics. Because of the social esteem associated with the product, the behaviour of other consumers affects individual preferences and hence consumption (see [48]). The esteem obtained decreases with the proportion of the population that buy the product and eventually esteem is no longer attached with the product when the product has become common. In fact, in this final stage disesteem could be attached to the non-labelled product whereas no esteem is attached to the eco-labelled product. The disesteem attached to the non-labelled product would increase as the number of compliers increases (see Fig 4 for a graphical exposition). Hence aggregate consumer demand is redirected toward the more socially salient product in the choice set.

Following this line of argument, the utility consumers gain from buying an eco-labelled product is divided into two parts. The first part is the functional utility which includes attributes such a taste and price. The second part is the supplementary utility associated with the eco-label. Supplementary utility includes altruism and “warm-glow” utility [49] gained from buying a good that has positive effects on the quality of life of others, on the natural environment or on animal welfare. In addition, consumers may gain supplementary utility from the esteem they gain from buying the good. [50] argues that because people care about status they care about other peoples’ perceptions of their preferences. Since preferences are unobservable, they use their actions as a signalling device for their preferences. To better understand this point, please see **Note 3** in S1 Appendix.

Relevant in our case is what happens to the non-altruistic effect when the price of a product with pro-social characteristics changes (i.e., this product becomes relatively more expensive). For consumers who value social reputation, the increase in the signalling value counteracts the effect of the price increase, in effect crowding-in reputational motives for buying [48]. Thus, if reputation is an important motive for buying goods with public good characteristics, then this reputation effect can lead to a reversal of reactions to changes in consumer prices/budgets (from that predicted by traditional price theory) as observed in our empirical results for the fair trade label. [51] report empirical evidence on reverse price reactions for the Danish milk market where organic milk enjoys a 30 percent market share. Analysis of scanner data on the effects of price discount weeks showed that the most reputation concerned consumers reduced demand (-6%) for organic milk during price discount week; the least concerned increased demand (+12%) and overall demand increased slightly (+3%). In addition, anecdotal evidence suggests that the organic label was losing market appeal in the first year of our time series. Much of this was driven by a public debate over whether organic food is actually healthier than conventional grown farm produce from a nutritional perspective (in this context, also see [52]). [53] find that UK markets for organic food purchases appear to be vulnerable to consumer dissatisfaction, particularly among heavy users of organic food products. Their results confirm the results of [54] that UK consumers of organic appear to change concerns and attitudes over time. This debate about the functional utility will also affect the esteem associated with the organic label. A drop in perceived quality of a product with the organic label will lead to a change in the aggregate demand for all categories of consumers. This model, therefore, offers another explanation for our empirical results.

## 5 Discussion

In this paper we look at the trends in consumer expenditure on eco-labelled grocery products during a recession when consumers face an income shock. We use supermarket loyalty card data for a range of food products sold under different eco-labels from a noted super market chain in the UK for our study. We employ data-driven methods to investigate how the recession impacted consumers’ aggregate purchase behaviour. Then we consider an econometric specification that looks at the difference, pre and post recession, in the purchases of various eco-labelled products (at a disaggregated level). We find that the share of consumer expenditure on fair trade products seems to hold up during the recession while the share of organic products seems to fall.

We now note a few points regarding our methodology. The regression specification that we use is simple, but it captures the effect that we want to model, *viz*., to see if there is a change between the baseline level of purchases that consumers undertake, for ethical reasons, pre-recession and the level of purchases that consumers make post recession. These purchases are measured as aggregate expenditure shares expended by consumers on eco-labelled categories. We note that for our setup, the *level* of expenditures on purchase of eco-labeled categories is not important. It is the *change* in the (total) expenditure amounts of eco-labeled categories purchased that is important. This point is informed by the theoretical models (and anecdotal evidence) informing our econometric approach. The models predict that *total* consumer expenditure on a category like, fair trade, for example, should stay the same. Therefore, we focus on changes in the *total* consumer expenditure for a eco-labelled category as a whole.

We note that our regression specification removes the effect of any trends or seasonality (assuming that these trends do not change by much over the two periods being differenced). We also note that in our specification we use two identical matching periods to difference expenditure shares for each eco-labelled category. In other words, if we have, say, higher periodic expenditures in December, pre-recession then differencing this with the matched December month post-recession is going to net away the effects of such periodicities. To better understand this point, please see the note in **Note 5** in S1 Appendix. We also note that our regression specification can also accommodate different trends for different eco-labeled categories. Since we consider a separate regression for each eco-labeled category—fair trade products, for example, can have a different trend than organic products.

However, our specification is not without its limitations. One drawback of our specification *vis-a-vis* identification will arise if the counterfactual expenditure trend (in the absence of the recession) was to change for a specific eco-labeled category, between the two periods being differenced (i.e, periods 1 and 3). Therefore, we have to *assume* that the counterfactual trend for a specific eco-labelled category would continue to be the same over the two periods being compared. We are not aware of any policies or sectoral changes in the time period that we consider in our analysis that might invalidate this assumption.

We also note that if we had detailed data over the entire time span of our analysis, *both* on the standard variant of a product and the eco-labeled variant of the same product, we could have teased out individual product effects using a difference-in-differences method (see for example, [55, 56] for examples of this approach). However, we do not have this data for all products in our data set. This is a limitation of our study which can be addressed in future research.

Despite these caveats, our study is important as it is one of the first studies to look at ethical purchases of eco-labelled products using market data in a recession. We note that there have been experimental studies in a laboratory environment looking at this question. In experiments we typically change some features of the choice environment and see the effect on subject choices. Crucial to this exercise is the ability to effectively vary the information set that is available to test subjects. In a laboratory setting it is not possible to alter many features of the choice environment and thus to effectively study how choices are made in a real world setting. We believe that the actual shopping behaviour of consumers is more indicative of consumer preferences (compared to an experimental setting) as these are revealed preferences in a real world setting taking into account real market trade-offs. We believe that market behaviour gives us a more accurate revelation of the underlying consumer preferences than when the subject behaviour is studied in an experimental setting.

The policy implications of our results can be considered from both a managerial as well as from a public (environmental) policy perspective. The implications of our work from a managerial perspective follow from the recent literature looking at the link between Corporate Social Responsibility (CSR) and the perception of a brand’s image. This literature suggest that CSR may help mould consumers’ impression of a brand and therefore CSR activities may affect various facets of brand performance [57, 58].

Our results are particularly in line with the managerial implications of findings reported in the marketing literature on brand performance during recessions –specifically the literature which finds empirical evidence to suggest that CSR initiatives during recessions provide a signal to customers of higher brand quality. For example, [59] show empirically that consumers associate product attributes that reflect concern about social and environmental issues with increased quality value during recessions. These authors point out that economic theory posits that changes in the relationship between how much consumers are willing to pay and their perception of the value they are receiving underpin behavioural changes (also see [60]). Particularly relevant to our context is their result which finds empirical evidence to suggest that CSR initiatives during recessions provide a signal to customers of higher brand quality (see [59]).

The management implication of our work stems directly from the fact that the use of eco-labels on products can be viewed as a manifestation of CSR. The use of labels (as with other CSR initiatives) provides a way of limiting the negative impact of recessions on brand performance. Even small changes in consumer demand can have a sizeable impact on brand profitability (see for example [60] who show that a consumer shift in demand as small as 1% point can severely dent firm profitability and alter market dynamics. In our specific case, the sales of fair trade products, for example, do not fall in a recession. In general, CSR activities, of which labelling is an instance, create a more favourable consumer perception of the brand. Therefore, when firms engage in these labelling schemes they align both social and managerial interests. Therefore the use of labelling schemes as a form of CSR can be a valuable tool for managers to insulate their brand during recessions while at the same time delivering a public good congruent with CSR values. Therefore, in times of economic recessions managers should maintain a focus on aspects that lead to consumer perceptions of higher quality and differentiation.

Even during stable economic periods managers may look at labelling schemes as a way to differentiate their brand and increase consumers’ confidence in the quality of the brand. Forward looking companies will recognize the crucial role of such ethical labelling schemes and invest in such initiatives to differentiate their brand. In so far as these schemes lead to consumer perceptions of higher brand quality and differentiation, esteemed brands may even be able to charge an even higher price for their products.

The question of what attributes that reflect concern about social and environmental issues mean to consumers also has implications with regard to policy-making. It is the challenge for policy-makers to understand how this meaning is socially constructed in a specific context and how to take this into account when environmental policies are developed, in particular.

The aspect of viewing labelling as a CSR tool for cushioning recessionary shocks also suggests subsequent avenues for future research such as looking at the financial performance, during recessions, of brands that engage in these ethical labelling schemes *vis-a-vis* brands which do not. Related to this point is the question of investigating to which extent companies increase ethical behaviour schemes (i.e, invest in CSR initiatives) during recessions to cushion themselves from the economic shock of the recession. Future research may also investigate whether the visibility of CSR activities makes a difference in or out of recession for firms that pro-actively engage in CSR initiatives (please see [59] for details).

## 6 Conclusion

Despite widespread academic interest, econometric analysis of supermarket retail data across sales of organic and fair trade grocery products is sparse. Our paper addresses this gap and is a first attempt at examining the effects on consumer purchase behaviour of eco-labelled (or “sustainable”) grocery products using market data under recessionary conditions. Specifically, this paper examines the effect of the recent recession on the observed expenditure shares of organic and fair trade products. We confirm empirically the trade reports about fair trade and organic sales during the economic recession and show that alternative models of consumer choice offer a rationale for these observations in the retail sector. Specifically, a model of context-dependent decision making (salience theory) and a model of moral motivation and social image both give an intuitive explanation for our empirical results. From a methodological perspective, our exploration of purchase behaviour at the consumer level provides insights for practitioners and for further research.

## Supporting information

S1 Appendix Notes [10, 18, 39, 42, 61]. (PDF)

S1 File Aggregate expenditure data. The data file aggspends.dta contains *weekly* expenditure for a period of 104 weeks (36 weeks in 2007, 52 weeks in 2008 and 16 weeks in 2009) on each major eco-labelled product category—organic, fair trade, carbon, carbon-fair trade and the category, “other”. This data is used in our exploratory graphical analysis.
(DTA)

S2 File Disaggregated consumer level expenditure data. The data file merge_newweekaggspends.dta contains disaggregated or individual consumer level data. This data is used in our econometric analysis.
(DTA)

## References

[1] Hamilton S, Zilberman D. Green markets, eco-certification, and equilibrium fraud. Journal of Environmental Economics and Management. 2006;52(3):627–633. doi: 10.1016/j.jeem.2006.05.002

[2] Buller H, Morris C. Growing goods: the market, the state, and sustainable food production. Environment and Planning A. 2004;36:1065–1084. doi: 10.1068/a35282

[3] Roe RE, Teisl MF, Deans CR. The economics of voluntary versus mandatory labels. Annual Review of Resource Economics. 2014;6:407–427. doi: 10.1146/annurev-resource-100913-012439

[4] Yokessa M, Marette SA. A Review of Eco-labels and their Economic Impact. International Review of Environmental and Resource Economics. 2019;13(1-2):119–163. doi: 10.1561/101.00000107

[5] Meis-Harris J, Klemm JC, Kaufman S, Curtis J. What is the role of eco-labels for a circular economy? A rapid review of the literature. Journal of Cleaner Production. 2021;306:127–134. doi: 10.1016/j.jclepro.2021.127134

[6] Nyborg K, Howarth RB, Brekke KA. Green consumers and public policy: On socially contingent moral motivation. Resource and Energy Economics. 2006;28(4):351–366. doi: 10.1016/j.reseneeco.2006.03.001

[7] Ibanez L, Grolleau G. Can Ecolabeling Schemes Preserve The Environment? Environmental and Resource Economics. 2008;40(2):233–249. doi: 10.1007/s10640-007-9150-3

[8] Mason CF. The Economics of Eco-Labeling: Theory and Empirical Implications. International Review of Environmental and Resource Economics. 2013;6:1–32. doi: 10.1561/101.00000054

[9] Segerson K. Voluntary Approaches to Environmental Protection and Resource Management. Annual Review of Resource Economics. 2013;5:161–180. doi: 10.1146/annurev-resource-091912-151945

[10] Fischer C, Lyon TP. A theory of multitier ecolabel competition. Journal of the Association of Environmental and Resource Economists. 2019;6(3):461–501. doi: 10.1086/702985

[11] Scott F, Sesmero JP. Market and welfare effects of quality perception in food labels. American Journal of Agricultural Economics. 2022;104:1747–1769. doi: 10.1111/ajae.12287

[12] Biswas A. A Study of Consumers’ Willingness to Pay for Green Products. Journal of Advanced Management Science. 2016;4(3):211–215. doi: 10.12720/joams.4.3.211-215

[13] VanDoorn J, Verhoef PC, Risselada H. Sustainability Claims and Perceived Product Quality: The Moderating Role of Brand CSR. Sustainability. 2020;12(9).

[14] Kortelainen M, Raychaudhuri J, Roussillon B. Effects of Carbon Reduction Labels: Evidence from Scanner Data. Economic Inquiry. 2016;54(2):1167–1187. doi: 10.1111/ecin.12278

[15] Carrigan M, de Pelsmacker P. Will ethical consumers sustain their values in the global credit crunch? Marketing Review. 2009;26(6):674–687. doi: 10.1108/02651330911001341

[16] Bondy T, Talwar V. Through Thick and Thin: How Fair Trade Consumers Have Reacted to the Global Economic Recession. Journal of Business Ethics. 2011;101(3):365–383. doi: 10.1007/s10551-010-0726-4

[17] Gilal FG, Zhang J, Paul J, Gilal NG. The role of self-determination theory in marketing science: An integrative review and agenda for research. European Management Journal. 2019;37(1):29–44. doi: 10.1016/j.emj.2018.10.004

[18] Bordalo P, Gennaioli N, Schleifer A. Salience and Consumer Choice. Journal of Political Economy. 2013;12(5):803–843. doi: 10.1086/673885

[19] Ariely D, Bracha A, Meier S. Doing good or doing well? Image motivation and monetary incentives in behaving prosocially. The American Economic Review. 2009;99(1):544–555. doi: 10.1257/aer.99.1.544

[20] Bénabou R, Tirole J. Identity, morals and taboos: Beliefs as assets. The Quarterly Journal of Economics. 2011;126(2):805–855. doi: 10.1093/qje/qjr002 22073409

[21] Grunert KG, Hieke S, Wills J. Sustainability labels on food products: Consumer motivation, understanding and use. Food Policy. 2014;44:177–189. doi: 10.1016/j.foodpol.2013.12.001

[22] De Bauw M, Franssens S, Vranken L. Trading off environmental attributes in food consumption choices. Food Policy. 2022;112:102338. doi: 10.1016/j.foodpol.2022.102338

[23] Rondoni A, Grasso S. Consumers behaviour towards carbon footprint labels on food: A review of the literature and discussion of industry implications. Journal of Cleaner Production. 2021;301:127031. doi: 10.1016/j.jclepro.2021.127031

[24] Poelman A, Mojet J, Lyon D, Sefa-Dedeh S. The influence of information about organic production and fair trade on preferences for and perception of pineapple, Food Quality and Preference. Food Quality and Preference. 2008;19:114–121. doi: 10.1016/j.foodqual.2007.07.005

[25] Zanolli R, Naspetti S. Consumer motives in the purchase of organic food. British Food Journal. 2002;104:643–653. doi: 10.1108/00070700210425930

[26] Bougherara D, Combris P. Eco-labelled food products: what are consumers paying for? European Review Agricultural Economics. 2009;36 (3):321–341. doi: 10.1093/erae/jbp023

[27] De Pelsmacker P, Driessen L, Rayp G. Do Consumers Care about Ethics? Willingness to Pay for Fairtrade Coffee. The Journal of Consumer Affairs. 2005;392:363–385. doi: 10.1111/j.1745-6606.2005.00019.x

[28] Foundation F. Annual Review: 2009-2010; 2010. Available from: https://www.fairtrade.org.uk/wp-content/uploads/legacy/doc/2009-2010.pdf.

[29] Soil Association Report. Organic Market Report 2009. Bristol: Soil Association; 2009.

[30] Deaton A, Muellbauer J. Economics and Consumer Behavior; 1980. doi: 10.1017/CBO9780511805653

[31] Kamakura WA, Du RY. How Economic Contractions and Expansions Affect Expenditure Patterns. Journal of Consumer Research. 2012;39:229–247. doi: 10.1086/662611

[32] Akerlof G, Kranton R. Economics and identity. Quarterly Journal of Economics. 2010;115(3):715–753. doi: 10.1162/003355300554881

[33] Dasgupta P, Southerton D, Ulph A, Ulph D. Consumer behaviour with environmental and social externalities: implications for analysis and policy. Environmental and Resource Economics. 2016;65(1):191–226. doi: 10.1007/s10640-015-9911-3

[34] Hastie T, Tibshirani R, Friedman J. The Elements of Statistical Learning: Data Mining, Inference, and Prediction. vol. Second Edition. Springer Series in Statistics; 2011.

[35] Hsiao C, Li Q, Racine JS. A consistent model specification test with mixed categorical and continuous data. Journal of Econometrics. 2007;140:802–826. doi: 10.1016/j.jeconom.2006.07.015

[36] Griffith R, O’Conell M, Smith K. Relative prices, consumer preferences, and the demand for food. Oxford Review of Economic Policy. 2015;31(1):116–130. doi: 10.1093/oxrep/grv004

[37] Dekimpe MG, Deleersnyder B. Business cycle research in marketing: a review and research agenda. Journal of the Academy of Marketing Science. 2018;46:31–58. doi: 10.1007/s11747-017-0542-9

[38] Dubé JP, Hitsch GJ, Rossi PE. Income and Wealth Effects on Private-Label Demand: Evidence from the Great Recession. Marketing Science. 2018;37(1):22–53. doi: 10.1287/mksc.2017.1047

[39] Hastings JS, Shapiro JM. Fungibility and consumer choice: Evidence from commodity price shocks. The Quarterly Journal of Economics. 2013;128(4):1449–1498. doi: 10.1093/qje/qjt018 26937053 PMC4771414

[40] Hauser JR, Wernerfelt B. An Evaluation Cost Model of Consideration Sets. The Journal of Consumer Research. 1990;16(4):393–408. doi: 10.1086/209225

[41] Dertwinkel-Kalt M, Lange MRJ, Koehler K, Wenzel T. Demand shifts due to salience effects: Experimental evidence. Journal of the European Economic Association. 2017;15(3):626–653. doi: 10.1093/jeea/jvw012

[42] Teyssier S, Etilé F, Combris P. Social-and self-images concerns in fair-trade. European Review of Agricultural Economics. 2015;42(4):579–606. doi: 10.1093/erae/jbu036

[43] Klockner CA, Ohms S. The importance of personal norms for purchasing organic milk. British Food Journal. 2009;111(11):1173–1187. doi: 10.1108/00070700911001013

[44] Saitone TL, Sexton RJ. Agri-food supply chain: evolution and performance with conflicting consumer and societal demands. European Review of Agricultural Economics. 2017;44:634–657. doi: 10.1093/erae/jbx003

[45] Griskevicius V, Tybur JM, VandenBergh B. Going Green to Be Seen: Status, Reputation, and Conspicuous Consumption. Journal of Personality & Social Psychology. 2010;98(3):392–404. doi: 10.1037/a0017346 20175620

[46] Richardson M, Stähler F. Fairtrade. Economic Record. 2014;90(291):447–461. doi: 10.1111/1475-4932.12136

[47] Dubé JP, Luo X, Fang Z. Self-Signalling and Prosocial Behavior: A Cause Marketing Mobile Field Experiment. Marketing Science. 2017;36(2):161–186. doi: 10.1287/mksc.2016.1012

[48] Brennan G, Pettit P. The Economy of Esteem. Oxford University Press; 2004.

[49] Andreoni J. Impure Altruism and Donations to Public Goods: A Theory of Warm-Glow Giving. The Economic Journal. 1990;100(401):464–477. doi: 10.2307/2234133

[50] Bernheim BD. A Theory of Conformity. Journal of Political Economy. 1994;102(5):841–77. doi: 10.1086/261957

[51] Kahsay GA, Andersen LM, Hansen LG. Price reactions when consumers are concerned about pro-social reputation. Frederiksberg: Department of Food and Resource Economics, University of Copenhagen; 2014. IFRO Working paper 2014/09.

[52] VanDoorn J, Verhoef PC. Willingness to pay for organic products: Differences between virtue and vice foods. International Journal of Research in Marketing. 2011;28(3):167–180. doi: 10.1016/j.ijresmar.2011.02.005

[53] Wier M, Jensen KO, Andersen LM, Millock K. The character of demand in mature organic food markets: Great Britain and Denmark compared. Food Policy. 2008;33:406–421. doi: 10.1016/j.foodpol.2008.01.002

[54] Weatherell C, Tregear A, Allinson J. In search of the concerned consumer: UK public perceptions of food, farming and buying local. Journal of rural studies. 2003;19(2):233–244. doi: 10.1016/S0743-0167(02)00083-9

[55] Muniba, Yu B. Does Innovative City Pilot Policy Stimulate the Chinese Regional Innovation: An Application of DID Model. International Journal of Environmental Research and Public Health. 2023;20(2). doi: 10.3390/ijerph20021245 36673993 PMC9859584

[56] Wang L, Muniba, Lakner Z, Popp J. The Impact of Water Resources Tax Policy on Water Saving Behavior. Water. 2023;15(5). doi: 10.3390/w15050916

[57] Guzman F, Davis D. The impact of corporate social responsibility on brand equity: consumer responses to two types of fit. Journal of Product and Brand Management. 2017;26(5):435–446. doi: 10.1108/JPBM-06-2015-0917

[58] Saxton GD, Gomez L, Ngoh Z, Lin YP, Dietrich S. Do CSR messages resonate? Examining public reactions to firms’ CSR efforts on social media. Journal of Business Ethics. 2019;155(2):359–377. doi: 10.1007/s10551-017-3464-z

[59] Bhattacharya A, Good V, Sardashti H. Doing good when times are bad: the impact of CSR on brands during recessions. European Journal of Marketing. 2020;54(9). doi: 10.1108/EJM-01-2019-0088

[60] Steenkamp JBEM, Heerde HJV, Geyskens I. What Makes Consumers Willing to Pay a Price Premium for National Brands over Private Labels? Journal of Marketing Research. 2010;47(6):1011–1024. doi: 10.1509/jmkr.47.6.1011

[61] Abeler J, Marklein F. Fungibility, Labels, and Consumption. Journal of the European Economic Association. 2017;15(1):99–127. doi: 10.1093/jeea/jvw007

