# Supplementary material for: Ecolabels and the economic recession
Source: PLoS One. 2023 Dec 4;18(12):e0294167. doi: 10.1371/journal.pone.0294167 (PMC10695377; doi:10.1371/journal.pone.0294167)
Supplement: S1 Appendix — (PDF) [file pone.0294167.s001.pdf]

S1 Appendix. Notes.

Note 1 To see how the salience model applies in our case, assume a choice set of an indivisible product – say coffee – that comes in two eco-labelled versions and is described by the attributes of public good quality (q) and price (p). Thus,in this choice set, we have two variants of the product denoted FT (fair trade) with (public good) quality q_{ft} and price p_{ft} and O (organic) with quality q_o and price p_o. For our case, we have $q_{ft} > q_o$ and $p_{ft} > p_o$. The consumer is fully informed about both attributes and evaluates both these products. An attribute (here either pro-social quality or price) is salient in the choice set if this attribute stands out relative to the other attributes. This means that each product in the choice set is compared to the reference product with average attributes of quality $\bar{q} = \frac{q_{ft}+q_o}{2}$ and price $\bar{p} = \frac{p_{ft}+p_o}{2}$. Salience will tilt consumer preferences toward the product with the highest quality price ratio. In our choice set of products FT and O, the public good quality of the product, or q, will be salient for both products if $\frac{q_{ft}}{p_{ft}} > \frac{\bar{q}}{\bar{p}}$ and price will be salient if $\frac{q_o}{p_o} > \frac{\bar{q}}{\bar{p}}$. This process then leads to a ranking of the choice set.

Note 2 A simple numerical example makes this idea clear. For simplicity we assume that our exercise is that of a discrete choice between two options only – FT (fair trade) or O (organic) of coffee. First let us consider a price change in free trade coffee only. Assume that before the price change $q_{ft} = 30$, $p_{ft} = 3$, $q_o = 20$ and $p_o = 5$. So $\bar{q} = \frac{q_{ft}+q_o}{2} = \frac{30+20}{2} = 25$ and $\bar{p} = \frac{p_{ft}+p_o}{2} = \frac{3+5}{2} = 4$. Now, $\frac{q_{ft}}{p_{ft}} = \frac{30}{3} = 10$, $\frac{q_o}{p_o} = \frac{20}{5} = 4$ and $\frac{\bar{q}}{\bar{p}} = \frac{25}{4} = 6.25$. Thus, $\frac{q_{ft}}{p_{ft}} > \frac{\bar{q}}{\bar{p}} > \frac{q_o}{p_o}$ – and so the public good quality is salient and FT is chosen since it is better along the public good quality dimension. Let us assume that the price of free trade coffee increases by 7 or $\Delta p_{ft} = 7$. Now, $q_{ft} = 30$, $p_{ft} = 10$, $q_o = 20$ and $p_o = 5$. So $\bar{q} = \frac{q_{ft}+q_o}{2} = \frac{30+20}{2} = 25$ and $\bar{p} = \frac{p_{ft}+p_o}{2} = \frac{10+5}{2} = 7.5$. Now, $\frac{q_{ft}}{p_{ft}} = \frac{30}{10} = 3$, $\frac{q_o}{p_o} = \frac{20}{5} = 4$ and $\frac{\bar{q}}{\bar{p}} \frac{25}{7.5} = 3.33$. Thus, $\frac{q_{ft}}{p_{ft}} < \frac{\bar{q}}{\bar{p}} < \frac{q_o}{p_o}$ – and so now the price is salient and O is chosen since it is better along the price dimension.

Now contrast this with an income change. Assume that before the income change we have the following configuration of public good quality and price : $q_{ft} = 30$, $p_{ft} = 10$, $q_o = 20$ and $p_o = 5$. So $\bar{q} = \frac{q_{ft}+q_o}{2} = \frac{30+20}{2} = 25$ and $\bar{p} = \frac{p_{ft}+p_o}{2} = \frac{10+5}{2} = 7.5$. Also, $\frac{q_{ft}}{p_{ft}} = \frac{30}{3} = 10$, $\frac{q_o}{p_o} = \frac{20}{5} = 4$ and $\frac{\bar{q}}{\bar{p}} = \frac{25}{7.5} = 3.33$. Thus, $\frac{q_{ft}}{p_{ft}} < \frac{\bar{q}}{\bar{p}} < \frac{q_o}{p_o}$ – and price is salient and O is chosen since it is better along the price dimension. Now assume that there is a negative income shock. If this consumer's earlier income was M, then a decline in this consumer's income to $M - \Delta$ leads to the same budget constraint and to the same utility-maximizing behaviour as in the situation where prices of both options FT and O each increase by Δ and income remains constant at M (because money is fungible and we assume discrete choice). Assume that $\Delta p_{ft} = \Delta_o = \Delta = 20$. Now, $q_{ft} = 10 + 20 = 30$, $p_{ft} = 30$, $q_o = 20$ and $p_o = 20 + 5 = 25$. So $\bar{q} = \frac{q_{ft}+q_o}{2} = \frac{30+20}{2} = 25$

and $\bar{p} = \frac{p_{ft}+p_o}{2} = \frac{30+25}{2} = 27.5$. Now, $\frac{q_{ft}}{p_{ft}} = \frac{30}{30} = 1$, $\frac{q_o}{p_o} = \frac{20}{25} = 0.8$ and $\frac{\bar{q}}{\bar{p}} = \frac{25}{27.5} = 0.9$. Thus, $\frac{q_{ft}}{p_{ft}} > \frac{\bar{q}}{\bar{p}} > \frac{q_o}{p_o}$ and so the public good quality is salient and FT is chosen since it is better along the public good quality dimension.

Note 3 To formalise this insight, consider a choice set as before of an indivisible eco-labelled product with two varieties: FT with (pro-social) quality q_{ft} and price p_{ft} and product O with quality q_o and price p_o with $q_{ft} > q_o$ and $p_{ft} > p_o$. Note that quality in this context refers to both the functional utility (taste etc.) and the supplementary utility associated with the eco-label (self-identity and social esteem). Quality of product i as perceived by an individual consumer can then be written as $q_i = w(v_i) + m(s, n_i, N_i)$ where v_i is the hedonic value of good i; s is the exogenous level of scrutiny; n_i denotes the consumer's belief of the moral standard, and N_i is the consumer's belief about the proportion of the population that buy the product (see [42]). Let consumers be distributed across some range $\mu[0,1]$ according to the density function $f(\mu)$ with utility $u = \mu(q-p)$.

We identify the consumer who is indifferent between buying the basic eco-labelled product and not buying it, with the condition : $WTP_O = 0$ which gives $\mu_o q_o - p_o = 0$ thus the preference level is : $\mu_o = \frac{p_o}{q_o}$. Similarly, we can identify the consumer who is indifferent between the two eco-labelled products, using the condition: $WTP_{O-FT} = 0$ which gives $\mu_{ft} q_{ft} - p_{ft} = \mu_{ft} q_o - p_o$ and leads to a preference level: $\mu_{ft} = \frac{p_{ft}-p_o}{q_{ft}-q_o}$.

If $f(\mu)$ is uniform on $[0,1]$ then $f(\mu) = 1$ and we have aggregate demand from three different groups of consumers ([10]) :

1. Those that do not buy an eco-labelled product : $D^0 = \frac{p_o}{q_o}$.
2. Those that buy the eco-labelled product O: $D^O = \frac{(p_{ft}-p_o)}{(q_{ft}-q_o)} - \frac{p_o}{q_o} = \frac{q_o - p_o q_{ft}}{q_o - p_o q_{ft}}$
3. Those that buy the eco-labelled product $FT : D^{FT} = 1 - \frac{(p_{ft}-p_o)}{(q_{ft}-q_o)} = \frac{(q_{ft}-q_o)-(p_{ft}-p_o)}{(q_{ft}-q_o)}$

It follows that a change in the price or in the perceived quality of product O will affect the aggregate demand of all three categories of consumers. In contrast, such changes for product FT will only affect demand for FT and O. In particular : $\frac{\partial D^{FT}}{\partial q_o} = \frac{p_{ft}-p_o}{(q_{ft}-q_o)^2}$ and $\frac{\partial D^O}{\partial q_{ft}} = \frac{p_o - p_{ft}}{(q_{ft}-q_o)^2}$.

Note 4 Before we conclude, we briefly discuss an interesting observation regarding the fungibility of income in our study which also stands in contrast to the neo-classical paradigm. Neo-classical households treat money as fungible. Fungibility implies that individual units of money allow substitution or interchangeability. One implication of the fungibility of income is that a consumer should choose the same commodity bundle if prices increase by a certain percentage as she chooses when income decreases by the same percentage. Consider a situation where we have a good that comes in several varieties that differ in quality and we have a consumer choosing just one indivisible unit of the good. If money is fungible, then the utility maximising consumer will choose the same good, whether she faces a decrease in income (income-decrease scenario) or a rise in prices (price-increase scenario). If money is not fungible then the consumer will substitute more towards the low-quality variety of the good in the price-increase scenario compared to the income-decrease scenario. Or equivalently, the consumer will substitute

(relatively) more towards the high-quality variety of the good in the income-decrease scenario. Laboratory experiments substantiated by theory (e.g., [61] and references therein) seem to indicate that consumers do indeed behave in a way that suggests that money is not fungible.

For our scenario, the non-fungibility of income during a recession implies that households will substitute towards the higher pro-social quality variant of the product or the fair trade varieties of a product. So fair trade shares will increase over time. Therefore, the results that we find in our paper appear to be in agreement with the idea that consumers do not consider income as fungible. We note, in this context, the work by [39] who test the fungibility of income using a panel data set on retail gasoline purchases in the US. They find that during the second half of 2008, during the financial recession, households in their data set substituted to higher-octane (better quality) gasoline. This is a result that mirrors our result in this paper. [39] reject the null hypothesis of fungiblity. In addition they use their data to test three different models that can account for their findings. This includes a salience model based on [18]. They make use of a rich data set comprising of panel microdata from a large U.S. grocery retailer with gasoline stations on site. They have transaction-level data on all gasoline and grocery purchases from 2006 to 2009 over 69 different retail locations all over the US. They focus on gas purchases and for each gasoline transaction, their data include the date, a store identifier, the number of gallons pumped, the grade of gasoline bought (regular, midgrade, or premium), and the amount paid for the transaction. While, like us, they use transaction level data (but on gasoline transactions), unlike us, their data set also has information on store identifiers and household identifiers linked to a retailer loyalty card. This rich data set allows them to construct a price series by store, grade, and date equal to the modal price across all gasoline transactions. They can also match transactions over time for a given household using the household identifier linked to the retailer loyalty card. So their data allows them to match gasoline transactions to grocery transactions by the same household. This is critical for testing the hypothesis of fungibility of income – that is whether households treat “gas money” as fungible with other income. They also have details of household income which is provided by the household to the retailer when applying for this loyalty card. They find that predictions from the model of category budgeting fits their data best and the fit of the other two models is limited. We have to leave such an exercise for future research as (unfortunately) the quality of our present dataset prevents us from carrying out such an exercise. We do not have (complete) data on any of the aforementioned variables, *viz*., store identifiers and household income – for reasons of data confidentiality. Moreover we only have transaction data on selected “ethical” products in our dataset (and their close substitutes) and only for transactions at the supermarket chain (this is a drawback of [39] as well that they only consider transactions from a single retailer).

Note 5 Let us consider a population where a certain fraction of the population displays "pro-social" behavior. We can ascribe multiple motives for such behavior, but we cannot identify these individual motives (at least with our current data). From the population of these individuals, people displaying altruistic or pro-social preferences record higher purchases of say fair trade, carbon-labeled, etc. products. Non-labeled variants of these products exist, but these consumers choose not to purchase these variants. These consumers are keen to incur expenditure on the labeled products because of their

altruistic or pro-social preferences. We posit that (and it follows from the theoretical models outlined in our text) it is the *total* expenditure on ethical consumption that is important for the consumer and not the money spent on individual products. It is the total expenditure on ethical products that increases consumers' utility. The recession provides an exogenous negative shock and so provides a "natural experiment" to study the effect on ethical consumption.

Also in this context, the *level* of expenditures on purchase of eco-labeled categories is not important. The *change* in the (total) expenditure amounts of eco-labeled categories purchased is important. We try to see if there is a change in purchases – between the baseline level of purchases that consumers undertake (for ethical reasons) pre-recession and the level of purchases that consumers make post recession.

Keeping in mind that is the changes in expenditure that is important, consider the example of an individual whose total expenditure on a certain eco-labelled category is £10, £8 and £30 for three months – October, November and December. We have a seasonality component built into these (hypothetical) numbers with much higher expenditures for December. Assume that these numbers are for the pre-recessionary period. After the recessionary effects have come into play, we have expenditures for the *same* three months (but for a different year) and for the same eco-labeled category, given by (say) £5, £4 and £15 – a 50 % cut in expenditure. Differencing over these two periods (which are matched one-for-one by month) nets out the effect of the recession on total expenditures for a eco-labeled category (other things unchanged). We do not need to de-seasonalise our data (like in a standard ARMA model, say, where the objective is to predict future values) as the differencing will show the effect of the recession on expenditures on eco-labeled goods.
